# Cognitive impairment in the elderly: the need for a comprehensive approach

**DOI:** 10.2478/abm-2022-0007

**Published:** 2022-04-29

**Authors:** Editorial Office of Asian Biomedicine

**Affiliations:** Faculty of Medicine, Chulalongkorn University, Bangkok 10330, Thailand

In the elderly, the spectrum of cognitive decline ranges from what can be classified as normal cognitive decline with aging or mild cognitive impairment to dementia. There are various factors associated with the risk of even mild cognitive impairment, including common chronic diseases, and lifestyle, environmental, and genetic factors.

Diabetes mellitus is well known to increase the risk of macro- and microvascular complications. Increased atherosclerosis a critical factor leading to vascular complications in patients with type 2 diabetes (T2DM). Metabolic derangements of T2DM include insulin resistance, hyperglycemia, and release of excess free fatty acids, and other metabolic abnormalities, which can damage the vascular wall, giving rise to endothelial dysfunction, platelet hyperactivity, oxidative stress, and low-grade inflammation [1]. Diabetes and obesity are among the modifiable risk factors for cognitive impairment in the elderly. Several overlapping neurodegenerative mechanisms, including oxidative stress, mitochondrial dysfunction, and inflammation have been identified in cognitive impairment. Products generated by chronic hyperglycemia and the receptor for advanced glycation end products (RAGE) provide crucial links between diabetes and cognitive decline. Other mechanisms linking diabetes and cognitive impairment include neuroinflammation, impaired neuronal plasticity, and other molecular mechanisms that link obesity, diabetes, and Alzheimer disease [2]. Moreover, there are some common biomarkers that are differentially expressed when compared with healthy controls both in patients with Alzheimer disease and in those with T2DM. These biomarkers might provide a useful way of screening T2DM patients to identify those at risk of developing Alzheimer disease [3].

In addition to T2DM, the elderly commonly develop dyslipidemia. Statins have been widely used to treat lipid abnormalities in this population with beneficial effects on the scores of the Mini-Mental State Exam scale in the short term (≤12 months). Moreover, statins could slow the deterioration of neuropsychiatric status and significantly improve activities of daily living ability in patients with Alzheimer disease, but did not show any advantage in changing Alzheimer's Disease Assessment Scale-cognitive scores [4]. A systematic review and meta-analysis of observational studies found no neurocognitive risk associated with statin treatment and suggests a potentially favorable role of statins in clinical settings [5]. In response to the Food and Drug Administration informational warnings about possible impairment of neurocognition associated with the use of statins, a systematic review of randomized trials suggests no adverse cognitive effects of statins, and recommended their use to reduce significant cardiovascular events [6]. At a cellular level, the effects of statins include impact on the cholesterol composition of the nerve and glial cell plasmalemma, neurotransmitter receptor mobilization, myelination, dendritic arborization of neurons, synaptic vesicle release, and cell viability [7]. Randomized clinical trials are required to explore this potential neuroprotective effect. In rodent models of Alzheimer disease, statins have been shown to have positive effects on amyloidosis [8]. Atorvastatin ameliorates behavioral measures of cognitive impairment, and downregulates Aβ1–42 production and tau hyperphosphorylation in the hippocampus and prefrontal cortex of amyloid precursor protein/presenilin 1 transgenic mice [9]. Encouraging effects of vitamin D, chondroitin, atorvastatin, and antihypertensive drugs, and adverse effects of antidepressants and benzodiazepines, may be important in affecting the rate of conversion to mild cognitive impairment and warrant further exploration in long-term studies [10].

In this issue, Kukula and Günaydın [11] report that atorvastatin significantly attenuated a deficit in the learned behavioral response to an adverse stimulus in an alloxan-induced murine model of diabetes mellitus. They were not able to elucidate the underlying mechanism for the reduced impairment in this model of memory and cognition. However, they were able to show that the protective effect might relate to mechanisms other than affecting glucose level. Their results suggest that management of diabetes and the use of atorvastatin to treat dyslipidemia could be beneficial to reduce neurogenerative disorder and subsequent cognitive decline.

Because there can be many factors associated with cognitive impairment, a comprehensive approach to prevent cognitive impairment in the elderly must be adopted. Age remains the strongest risk factor for cognitive impairment and dementia. The incidence of cognitive impairment increases exponentially with each decade. In addition, the elderly frequently have vascular conditions such as hypertension, diabetes, dyslipidemia, and obesity, which are linked to cognitive decline, and need to be managed. The presence of cardiovascular risks is strongest when documented in midlife, rather than late life, and when multiple factors are present. Lifestyle factors such as smoking, alcohol consumption, sedentary habits, and social isolation are also important. Other factors that might increase the risk of dementia include chronic kidney disease, depression, head trauma, hearing loss, exposure to certain medications and toxins, and obstructive sleep apnea and other medical illnesses [12].

A comprehensive assessment of the modifiable causes of dementia is necessary. Treatment of modifiable causes of dementia can change the quality of life of the elderly. Individual therapeutic interventions may make only small changes to the quality of life of individuals afflicted with dementia, but when used in combination, these interventions may synergize and potentially affect long-term outcome [12, 13]. Given the problem of dementia care on a societal scale, in economic and sociological terms the overall impact of such therapies may be substantial. Presently there are no disease-modifying treatments for any of the neurodegenerative dementias. Instead, clinicians have limited therapeutic tools to reduce the cognitive and behavioral harm of dementia. Judicious use of statins holds promise.
